# Independent linear association between flow-mediated dilation and predicted intermediate-to-high 10-year ASCVD risk in patients with type 2 diabetes: a retrospective study

**DOI:** 10.3389/fendo.2026.1986597

**Published:** 2026-10-14

**Authors:** Hou KJ, Shen SY, Zong CY, Wang AY, Hu Y, Song YX, Wu XH

**Affiliations:** 1 The Second Clinical Medical College, Zhejiang Chinese Medical University, Hangzhou, China; 2 Tongxiang Hospital of Traditional Chinese Medicine, Tongxiang, Zhejiang, China; 3 Hangzhou Normal University, Hangzhou, China; 4 Geriatric Medicine Center, Key Laboratory of Endocrine Gland Diseases of Zhejiang Province, Department of Endocrinology, Zhejiang Provincial People’s Hospital (Affiliated People’s Hospital, Hangzhou Medical College), Hangzhou, Zhejiang, China

**Keywords:** ASCVD risk, China-PAR model, endothelial dysfunction, flow-mediated dilation, type 2 diabetes

## Abstract

**Background:**

Vascular endothelial dysfunction precedes atherosclerosis in type 2 diabetes (T2DM). However, the specific nature of the association between flow-mediated dilation (FMD) and the intermediate-to-high 10-year atherosclerotic cardiovascular disease (ASCVD) risk remains to be fully elucidated. This study aimed to investigate this correlation in a large sample of Chinese patients with T2DM.

**Methods:**

We retrospectively analyzed 1,272 T2DM patients managed at the National Metabolic Management Center (MMC) between March 2020 and March 2025. Endothelial function was assessed via brachial artery FMD (dysfunction: FMD < 6.5%). Multivariate linear and logistic regressions identified independent factors. Restricted cubic spline (RCS) models assessed non-linear associations between FMD and the intermediate-to-high 10-year ASCVD risk.

**Results:**

Overall, 78.9% (n=1,003) exhibited endothelial dysfunction. Multivariate analysis identified older age, higher systolic blood pressure, male sex, smoking, hypertension, and the intermediate-to-high ASCVD risk were independently associated with impaired FMD (all *p* < 0.05). FMD showed an independent inverse association with intermediate-to-high 10-year ASCVD risk (OR = 0.90, 95% CI: 0.82-0.99, *p* < 0.05) and moderate discriminative ability (AUC = 0.700, 95% CI: 0.66–0.75). Crucially, RCS analysis confirmed a linear negative correlation between FMD and the intermediate-to-high ASCVD risk (*p*-overall = 0.004), with no evidence of non-linearity (*p*-non-linear = 0.653).

**Conclusions:**

Endothelial dysfunction is highly prevalent in T2DM. FMD was independently, inversely and linearly associated with the intermediate-to-high calculated 10-year ASCVD risk.

## 1 Introduction

Type 2 diabetes mellitus (T2DM) is a complex systemic metabolic disorder affecting multiple organ systems. Vascular complications represent the most prevalent chronic sequelae of T2DM and constitute the leading cause of diabetes-related mortality and disability (1). The vascular endothelium serves as the primary barrier maintaining vascular homeostasis, orchestrating critical physiological functions including the regulation of vascular tone, inhibition of thrombosis, and modulation of inflammatory and oxidative stress responses (2). Accumulating evidence indicates that endothelial dysfunction is not merely a consequence but a pivotal pathophysiological initiator of diabetic vasculopathy and atherosclerosis (3).

Brachial artery flow-mediated dilation (FMD), a non-invasive surrogate marker predominantly mediated by nitric oxide (NO), reliably reflects endothelium-dependent vasodilation and correlates well with coronary endothelial function (4). Numerous prospective studies have established that impaired FMD is an independent predictor of adverse cardiovascular outcomes, including coronary artery disease, ischemic stroke, and heart failure (5–7). However, the specific quantitative association—particularly the potential non-linear dynamics—between FMD impairment and the 10-year predicted risk of atherosclerotic cardiovascular disease (ASCVD) in patients with T2DM has not been fully elucidated.

Given that 10-year ASCVD risk scores are the cornerstone of current clinical guidelines for risk stratification and therapeutic decision-making (8), clarifying the relationship between this early subclinical marker (FMD) and established risk prediction models is essential for optimizing early intervention strategies. Therefore, this study aims to investigate the correlation, cross-sectional association, and dose-response relationship between FMD and intermediate-to-high 10-year ASCVD risk in a large cohort of patients with T2DM.

## 2 Materials and methods

### 2.1 Study design and participants

This retrospective study enrolled 1,272 patients with T2DM who visited the Department of Endocrinology at Zhejiang Provincial People’s Hospital and were managed under the standardized protocol of the National Metabolic Management Center (MMC) between March 2020 and March 2025. The study protocol was approved by the Ethics Committee of Zhejiang Provincial People’s Hospital (Approval No. ZRYL-2023-Other-350) and conducted in accordance with the Declaration of Helsinki. Written informed consent was obtained from all participants.

### 2.2 Inclusion and exclusion criteria

Inclusion criteria were: (1) diagnosis of T2DM based on the 2025 American Diabetes Association (ADA) standards (9); (2) age >18 years; and (3) complete clinical data including FMD and 10-year ASCVD risk assessment.

Exclusion criteria included: (1) other types of diabetes (e.g., type 1 diabetes, latent autoimmune diabetes in adults, specific types, or gestational diabetes); (2) presence of severe acute infections, active autoimmune diseases, or malignancies; and (3) missing data for key variables (FMD, ASCVD risk score, or major laboratory parameters) (**Figure 1**).

**Figure 1:**
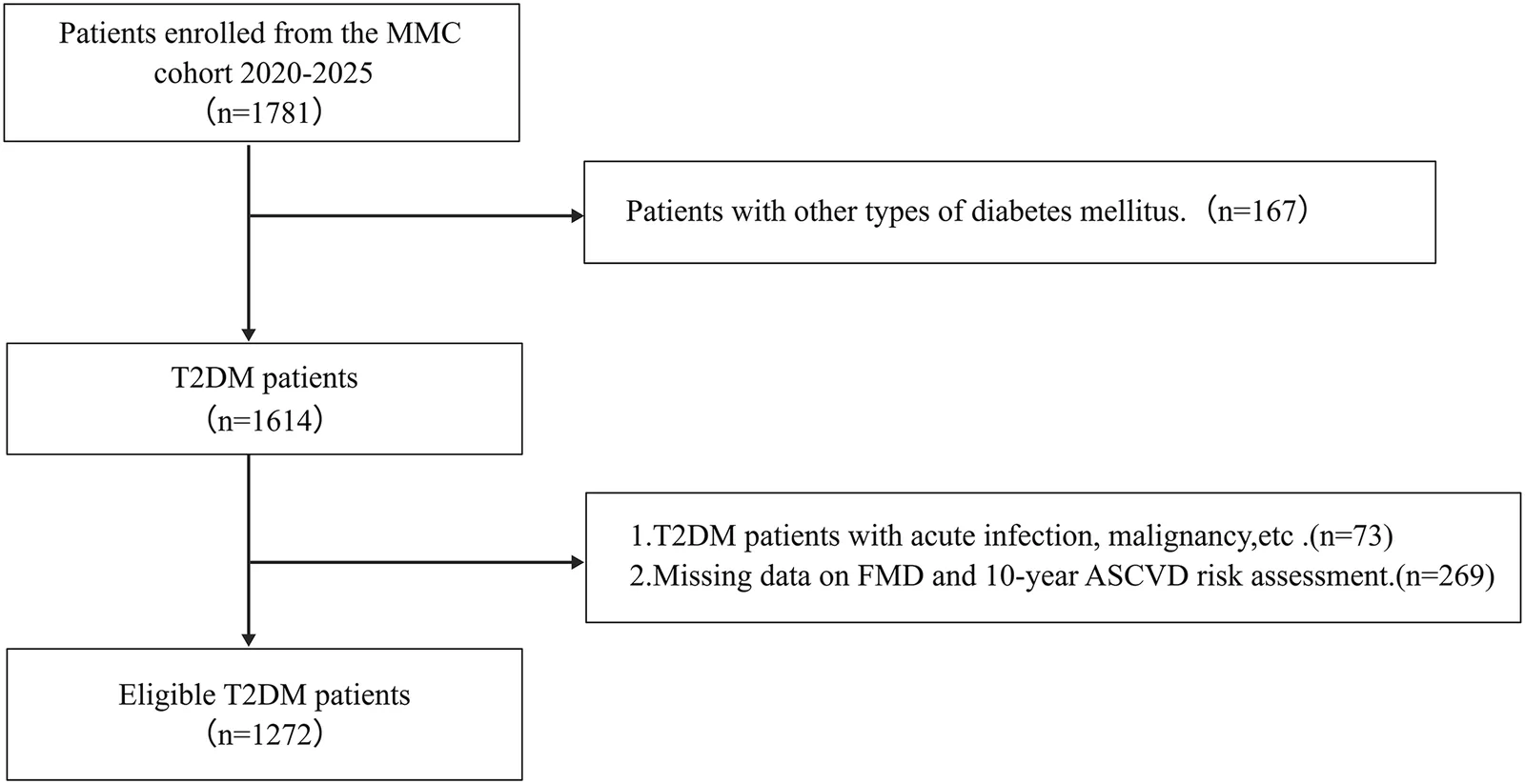
Flow chart of participant screening and enrollment.

### 2.3 Data collection and anthropometric measurements

Demographic data (age, sex), diabetes duration, lifestyle factors (smoking and alcohol consumption), and medical history (hypertension, dyslipidemia, coronary artery disease, stroke, peripheral artery disease, fatty liver) were collected via electronic medical records. Smoking was defined as current smoking; former and never smokers were classified as non-smokers; alcohol consumption was defined as regular drinking over a prolonged period.

Anthropometric measurements included height, weight, waist circumference, hip circumference, heart rate, and blood pressure (systolic and diastolic), measured by trained nurses using standardized protocols. Body mass index (BMI) and waist-to-hip ratio (WHR) were calculated.

### 2.4 Laboratory assessments

After an overnight fast of at least 8 hours, venous blood and morning urine samples were collected. Biochemical parameters were measured using standard enzymatic colorimetric methods, including: fasting plasma glucose (FPG), 2-hour postprandial glucose (2h-PG), glycated hemoglobin (HbA1c), fasting insulin, C-peptide, total cholesterol (TC), triglycerides (TG), high-density lipoprotein cholesterol (HDL-C), low-density lipoprotein cholesterol (LDL-C), apolipoprotein A-1 (ApoA-1), apolipoprotein B (ApoB), creatinine (Cr), blood urea nitrogen (BUN), uric acid (UA), homocysteine (Hcy), and free fatty acids (FFA). Thyroid function, complete blood count, and urinary albumin-to-creatinine ratio (UACR) were also assessed.

The homeostasis model assessment of insulin resistance (HOMA-IR) and β-cell function (HOMA-β) (10) were calculated using standard formulas. The estimated glomerular filtration rate (eGFR) was derived using the CKD-EPI equation (11).

### 2.5 Assessment of endothelial function (FMD)

Brachial artery FMD was assessed using a high-resolution color Doppler ultrasound system (probe frequency ≥7.5 MHz) by experienced sonographers blinded to clinical data (12). Participants rested in a supine position for 10 minutes in a quiet, temperature-controlled room (22–24 °C) after an 8-hour fast. All vasoactive medications were withheld for at least 24 hours prior to the examination.

A blood pressure cuff was placed on the forearm distal to the olecranon process. The brachial artery diameter was measured at end-diastole in the resting state. The cuff was then inflated to 50 mmHg above systolic blood pressure (or ≥200 mmHg) for 5 minutes to induce ischemia, followed by rapid deflation. The peak brachial artery diameter was recorded within 60 seconds post-deflation. FMD was calculated as: FMD (%)=(Peak diameter−Baseline diameter)/Baseline diameter×100%. Endothelial dysfunction was defined as FMD < 6.5% (4). For subgroup analysis, participants were stratified into quartiles based on FMD values: Q1 (0.1%–2.8%), Q2 (2.9%–4.3%), Q3 (4.4%–6.1%), and Q4 (6.2%–14.4%).

### 2.6 Assessment of 10-year ASCVD risk

The 10-year risk of ASCVD was estimated using the China-PAR (Prediction for ASCVD Risk in China) project equation (Prediction for ASCVD Risk in China), which is validated for the Chinese population. This model incorporates age, sex, geographical region, urban/rural status, smoking, diabetes, hypertension, family history of premature CVD, waist circumference, HDL-C, and non-HDL-C. The 10-year risk of ASCVD was assessed using the China-PAR prediction model (13). Patients with an estimated 10-year ASCVD risk ≥5% were categorized into the intermediate-to-high risk group (14).

### 2.7 Assessment of diabetic chronic complications

Vascular stiffness was evaluated by measuring brachial-ankle pulse wave velocity (baPWV) using an automated arteriosclerosis detector (BP-203RPEIII, Omron, Japan). The ankle-brachial index (ABI) was measured to screen for peripheral artery disease. Carotid intima-media thickness (CIMT) and plaques were assessed via color Doppler ultrasound (EPIQ7, Philips, Netherlands). Diabetic peripheral neuropathy was diagnosed using a six-channel electromyography system (KeyPoint, Medtronic). Diabetic retinopathy was graded by certified ophthalmologists based on fundus examination findings.

### 2.8 Statistical analysis

Normality of continuous variables was assessed using the Kolmogorov-Smirnov test. Normally distributed data are presented as mean ± standard deviation (SD), while non-normally distributed data are expressed as median (interquartile range, IQR). Categorical variables are reported as frequencies (percentages).

Differences among the four FMD quartile groups were compared using one-way analysis of variance (ANOVA) for normally distributed variables, Kruskal-Wallis H test for non-normal variables, and Chi-square test for categorical variables. Univariate and multivariate linear regression analyses were performed to identify factors independently associated with FMD. Multivariate logistic regression analysis was conducted to evaluate the independent association between FMD and intermediate-to-high 10-year ASCVD risk, reporting odds ratios (ORs) with 95% confidence intervals (CIs).

The discriminative ability of FMD for intermediate-to-high ASCVD risk was assessed using receiver operating characteristic (ROC) curves, and the area under the curve (AUC) and its 95% confidence interval were calculated using DeLong’s method. Restricted cubic spline (RCS) models with three knots (located at the 10th, 50th, and 90th percentiles) were employed to visualize and test the potential non-linear dose-response relationship between FMD and intermediate-to-high 10-year ASCVD risk. The RCS model was adjusted for age, sex, SBP, hypertension and smoking, consistent with the multivariate logistic regression model.

All statistical analyses were performed using SPSS version 25.0 (IBM Corp., Armonk, NY, USA) and R software version 4.2.0 (R Foundation for Statistical Computing, Vienna, Austria) for RCS modeling. A two-sided *p* < 0.05 was considered statistically significant.

## 3 Results

### 3.1 Baseline characteristics of the study population

A total of 1,272 patients with T2DM were included in the final analysis, comprising 879 men (69.1%) and 393 women (30.9%). The overall prevalence of endothelial dysfunction was 78.9% (n = 1,003).

As shown in **Table 1**, patients in the lower FMD quartiles were significantly older, had a longer duration of diabetes, higher systolic blood pressure (SBP), and greater waist circumference and visceral-to-subcutaneous fat ratios compared to those in the higher quartiles (all *p* < 0.05). The proportions of males, current smokers, alcohol consumers, and patients with hypertension increased significantly across decreasing FMD quartiles (all *p* < 0.05). No significant differences were observed among the groups regarding diastolic blood pressure, BMI, subcutaneous fat area, or the prevalence of dyslipidemia, hyperuricemia, coronary artery disease, stroke, peripheral artery disease, and fatty liver disease (all *p* > 0.05).

**Table 1:** Baseline characteristics by FMD quartiles.

Variables	Q1 (0.1-2.8)	Q2 (2.9-4.3)	Q3 (4.4-6.1)	Q4 (6.2-14.4)	*p*
Age, years	55.00 (47.00,63.00)	54.00 (43.75,62.00)	52.00 (42.00,59.75)	44.00 (37.00,55.00)	<.001
Male sex, n (%)	239 (76.85)	219 (73.99)	233 (67.34)	188 (58.93)	<.001
BMI, kg/m²	25.06 (22.86,27.48)	24.77 (22.81,27.45)	25.06 (22.75,27.72)	24.39 (22.14,27.55)	0.432
SBP, mmHg	132.00 (121.00,143.50)	130.00 (121.00,140.50)	127.00 (118.00,138.00)	127.00 (117.00,137.00)	<.001
DBP, mmHg	79.00 (72.00,87.00)	79.00 (73.00,87.00)	78.00 (72.00,85.00)	79.00 (72.00,86.00)	0.270
Hypertension, n (%)	162 (52.77)	137 (46.76)	116 (34.12)	68 (21.45)	<.001
Smoking, n (%)	158 (51.30)	131 (44.56)	135 (39.59)	98 (30.91)	<.001
Drinking, n (%)	144 (46.75)	131 (44.56)	139 (40.76)	113 (35.65)	0.025
Disease duration, months	57.50 (5.75,128.00)	36.00 (3.00,95.25)	36.00 (5.00,120.00)	24.00 (2.00,84.00)	<.001
WC, cm	93.00 (86.00,101.00)	93.00 (87.00,99.00)	92.00 (85.00,98.00)	91.00 (85.00,99.00)	0.044
VFA, cm²	100.00 (73.25,122.00)	103.95 (78.00,128.00)	94.00 (73.00,121.10)	93.00 (65.00,126.50)	0.098
SFA, cm²	168.50 (135.25,212.50)	173.00 (136.55,217.00)	172.00 (136.00,220.25)	168.25 (131.25,216.00)	0.614
Visceral-to-Subcutaneous fat ratio	0.57 (0.48,0.67)	0.57 (0.47,0.66)	0.54 (0.46,0.64)	0.54 (0.43,0.67)	0.010
Hyperlipidemia, n (%)	128 (41.56)	109 (37.20)	130 (38.24)	109 (34.38)	0.322
Hyperuricemia, n (%)	43 (13.96)	49 (16.72)	53 (15.59)	42 (13.25)	0.616
CAD, n (%)	8 (2.60)	9 (3.07)	7 (2.06)	7 (2.21)	0.852
Stroke, n (%)	8 (2.60)	8 (2.73)	5 (1.47)	1 (0.32)	0.076
PAD, n (%)	8 (2.60)	8 (2.74)	7 (2.06)	5 (1.58)	0.752
NAFLD, n (%)	116 (37.66)	123 (41.98)	130 (38.12)	119 (37.54)	0.638

Data are given as number (percentage) for categorical variables and median (inter-quar-tile range) for continuous variables. *p*-values < 0.05 were considered statistically signific-ant. BMI, body mass index; SBP, systolic blood pressure; DBP, diastolic blood pressure; HTN, hypertension; WC, waist circumference; VFA, visceral fat area; SFA, subcutaneous fat area; HLP, hyperlipidemia; HUA, hyperuricemia, CAD, coronary artery disease; PAD, peripheral artery disease; NAFLD, non-alcoholic fatty liver disease.

### 3.2 Comparison of laboratory parameters across FMD quartiles

Laboratory assessments revealed a gradient effect across FMD quartiles (**Table 2**). Lower FMD levels were significantly associated with elevated levels of blood urea nitrogen (BUN), serum creatinine (Cr), homocysteine (Hcy), and urinary albumin-to-creatinine ratio (UACR), as well as reduced estimated glomerular filtration rate (eGFR) and platelet count (Plt) (all *p* < 0.05). Furthermore, significant differences were observed in free fatty acids (FFA), total cholesterol (TC), low-density lipoprotein cholesterol (LDL-C), and free triiodothyronine (FT3) across the groups (all *p* < 0.05). Conversely, no statistically significant differences were found in fasting plasma glucose, 2-hour postprandial glucose, HbA1c, insulin, C-peptide, liver function tests, triglycerides, or HDL-C levels (all *p* > 0.05).

**Table 2:** Laboratory parameters by FMD quartiles.

Variables	Q1 (0.1-2.8)	Q2 (2.9-4.3)	Q3 (4.4-6.1)	Q4 (6.2-14.4)	*p*
FBG, mmol/L	6.58 (5.55,7.83)	6.64 (5.56,8.17)	6.50 (5.44,7.88)	6.66 (5.71,8.00)	0.504
2h-PG, mmol/L	14.93 (12.37,18.08)	15.96 (11.70,19.03)	14.89 (11.23,18.20)	15.07 (11.17,17.72)	0.095
FINS, µIU/ml	6.37 (3.55,11.26)	5.76 (3.10,10.04)	6.12 (2.75,10.84)	7.30 (3.29,11.01)	0.225
2h-INS, µIU/ml	30.17 (18.77,51.34)	27.12 (16.10,50.86)	32.05 (16.63,53.05)	32.97 (17.35,60.32)	0.089
F-C-P, ng/ml	1.64 (1.02,2.31)	1.74 (1.15,2.42)	1.63 (1.01,2.46)	1.86 (1.25,2.46)	0.074
2h-C-P, ng/ml	5.39 (3.72,7.64)	5.55 (3.87,7.57)	5.42 (3.82,7.52)	5.67 (3.80,7.93)	0.806
HOMA-IR	2.03 (0.94,3.64)	1.74 (0.84,3.18)	1.75 (0.81,3.64)	2.01 (0.96,3.57)	0.312
HOMA-β	41.64 (21.23,80.37)	37.09 (20.01,67.95)	36.91 (19.29,70.83)	41.67 (19.56,77.81)	0.346
HbA1c, %	8.60 (7.25,10.60)	8.50 (6.90,10.45)	8.50 (6.90,10.50)	8.60 (6.80,10.55)	0.630
Hb, g/L	146.00 (135.00,155.75)	146.00 (134.00,156.00)	147.00 (133.75,157.00)	145.00 (132.25,157.00)	0.748
RBC, *10^12/L	4.80 (4.43,5.14)	4.80 (4.48,5.20)	4.81 (4.47,5.20)	4.83 (4.50,5.21)	0.795
WBC, *10^9/L	6.30 (5.30,7.50)	6.58 (5.51,7.77)	6.35 (5.38,7.30)	6.41 (5.25,7.53)	0.244
PLT, *10^9/L	206.00 (171.00,243.00)	210.00 (177.75,247.25)	216.00 (185.00,248.00)	219.00 (188.00,260.00)	0.010
ALT, U/L	23.00 (17.00,34.00)	23.00 (17.00,37.00)	26.00 (17.00,40.75)	26.00 (17.00,41.00)	0.078
AST, U/L	21.00 (18.00,29.00)	22.00 (17.00,30.00)	23.00 (18.00,31.00)	23.00 (18.00,33.00)	0.097
γ-Gt, U/L	28.00 (18.00,44.00)	28.00 (19.00,51.25)	27.00 (18.00,47.00)	28.00 (18.00,46.00)	0.794
Hcy, µmol/L	12.60 (10.70,14.50)	12.55 (10.70,14.90)	12.50 (10.38,14.40)	11.30 (9.70,13.70)	<.001
BUN, mmol/L	5.53 (4.65,6.58)	5.41 (4.63,6.51)	5.23 (4.29,6.23)	4.72 (4.01,5.77)	<.001
Cr, µmol/L	73.30 (62.75,82.65)	72.80 (61.75,84.15)	72.75 (60.30,82.50)	66.50 (54.60,79.95)	<.001
UA, µmol/L	342.00 (285.00,395.75)	348.00 (278.00,418.50)	338.00 (268.00,402.50)	328.00 (266.00,401.00)	0.277
eGFR, ml/min/1.73 m²	100.02 (88.22,112.08)	100.72 (87.81,114.85)	101.95 (88.55,116.88)	110.27 (95.30,126.55)	<.001
UACR, mg/g	13.46 (7.10,43.24)	12.57 (7.69,28.83)	11.44 (7.15,23.06)	10.31 (6.43,25.35)	0.009
APOA-1, g/L	1.18 (1.04,1.34)	1.19 (1.06,1.34)	1.17 (1.05,1.34)	1.19 (1.03,1.33)	0.929
APOB, g/L	0.88 (0.70,1.05)	0.88 (0.72,1.03)	0.87 (0.72,1.04)	0.90 (0.74,1.07)	0.625
FFA, µmol/L	426.00 (283.75,593.25)	482.00 (312.50,638.50)	435.50 (311.25,602.50)	487.00 (361.50,636.00)	0.005
TG, mmol/L	1.39 (0.93,2.06)	1.47 (0.99,2.32)	1.40 (0.95,2.14)	1.43 (1.01,2.12)	0.683
TC, mmol/L	4.71 (3.83,5.39)	4.79 (3.89,5.47)	4.59 (3.78,5.24)	4.80 (4.11,5.51)	0.029
HDL-c, mmol/L	1.02 (0.85,1.19)	1.00 (0.87,1.19)	1.00 (0.88,1.16)	1.01 (0.88,1.21)	0.968
LDL-c, mmol/L	2.61 (2.05,3.36)	2.78 (2.12,3.35)	2.63 (2.08,3.24)	2.79 (2.31,3.37)	0.039
fT3, ng/L	3.17 (2.92,3.50)	3.27 (3.00,3.59)	3.21 (2.93,3.53)	3.31 (3.05,3.61)	0.009
fT4, ng/L	9.29 (8.36,10.33)	9.33 (8.40,10.74)	9.33 (8.33,10.41)	9.33 (8.39,10.43)	0.691
TSH, mIU/L	1.64 (1.10,2.46)	1.62 (1.11,2.31)	1.67 (1.09,2.38)	1.70 (1.09,2.55)	0.867

*p*-values < 0.05 were considered statistically significant.

FBG, fasting blood-glucose; 2h-PG, 2-hour postprandial plasma glucose; FINS, fasting insuli-n; 2h-INS, 2-hour postprandial ins-ulin; F-C-P, fasting C-peptide; 2h-C-P, 2-hour postprand-ial C-peptide; HOMA-IR, homeostas-is model assessment of insulin resistance; HOMA-β, homeostasis model assessment of β-cell function; HbA1c, glycated haemoglobin; Hb, hemog-lobin; RBC, red blood cell count; WBC, white blood cell count; PLT, platelet count; ALT, alanine aminotransferase; AST, aspartate aminotransferase; γ-Gt, gamma-glutamyl transferase; Hcy, homocysteine; eGFR, estimated glomerular filtration rate; BUN, blood urea nitrogen; Cr, creatinine; UA, uric acid; UACR, urinary albumin-to-creatinine ratio; APOA-1, apolip-oprotein A-1; APOB, apolipoprotein B; FFA, free fatty acids; TG, triglycerides; TC, total cholesterol; HDL-c, high-density lipoprotein-cholesterol; LDL-c, low-density lipoprotein-cholesterol; fT3, free triiodothyronine; fT4, free thyroxine; TSH, thyroid-stimulating hormone.

### 3.3 Diabetic complications and 10-year ASCVD risk stratification

The prevalence of diabetic complications and cardiovascular risk profiles varied significantly by FMD status (**Table 3**). Decreasing FMD levels were associated with increased brachial-ankle pulse wave velocity (baPWV), decreased left ventricular ejection fraction (LVEF), and increased ankle-brachial index (ABI) (all *p* < 0.05). The prevalence of carotid plaques, microalbuminuria, and diabetic peripheral neuropathy rose significantly in the lower FMD quartiles (all *p* < 0.05). Notably, the proportion of patients classified as intermediate-to-high risk for 10-year ASCVD (≥5%) increased markedly from Q4 to Q1 (all *p* < 0.05). However, the prevalence of diabetic retinopathy did not differ significantly among the groups (*p*>0.05).

**Table 3:** Prevalence of complications and intermediate-to-high ASCVD risk by FMD quartiles.

Variables	Q1 (0.1-2.8)	Q2 (2.9-4.3)	Q3 (4.4-6.1)	Q4 (6.2-14.4)	*p*
ABI	1.14 (1.05,1.19)	1.12 (1.06,1.20)	1.11 (1.04,1.18)	1.10 (1.03,1.17)	0.001
baPWV, cm/s	1523.50 (1369.38,1749.88)	1533.50 (1361.25,1721.25)	1443.50 (1300.00,1638.50)	1377.00 (1250.00,1573.50)	<.001
LVEF, %	65.00 (61.50,68.00)	65.00 (61.00,68.00)	65.00 (62.00,68.00)	65.00 (63.00,69.00)	0.024
DR, n (%)	65 (26.42)	57 (23.95)	70 (25.36)	49 (18.35)	0.129
MAU, n (%)	91 (29.26)	68 (22.97)	65 (18.79)	67 (21.00)	0.011
DN, n (%)	209 (71.82)	178 (62.68)	193 (59.94)	165 (54.64)	<.001
carotid plaque, n (%)	174 (56.86)	147 (51.22)	161 (47.35)	99 (31.43)	<.001
10-year ASCVD risk					
low group, n (%)	17 (5.47)	24 (8.11)	37 (10.69)	82 (25.71)	<.001
Intermediate-to- high group, n (%)	294 (94.53)	272 (91.89)	309 (89.31)	237 (74.29)	<.001

*p*-values < 0.05 were considered statistically significant.

ABI, ankle-brachial index; baPWV, brachial-ankle pulse wave velocity; LVEF, left ventricul-ar ejection fraction; DR, diabetic retinopathy; MAU, microalbuminuria; DN, diabetic nephropa-thy; 10-year ASCVD risk, 10-year risk of atherosclerotic cardiovascular disease.

### 3.4 Correlates of endothelial dysfunction in T2DM

Univariate linear regression analysis identified several factors negatively correlated with FMD, including age, diabetes duration, SBP, waist circumference, BUN, Cr, UACR, baPWV, presence of carotid plaques, microalbuminuria, peripheral neuropathy, intermediate-to-high 10-year ASCVD risk score, male sex, smoking, alcohol consumption, and hypertension (all *p* < 0.05). Positive correlations were observed with Plt, eGFR, FFA, and LVEF (all *p* < 0.05) (**Table 4**).

**Table 4:** Univariate linear regression analysis for FMD.

Variables	β	S.E	t	*p*	β (95%CI)
Age, years	-0.05	0.01	-10.64	<.001	-0.05 (-0.07 ~ -0.04)
Male sex	-1.00	0.14	-6.98	<.001	-1.00 (-1.28 ~ -0.72)
Smoking	-0.88	0.14	-6.52	<.001	-0.88 (-1.15 ~ -0.62)
Drinking	-0.53	0.14	-3.88	<.001	-0.53 (-0.80 ~ -0.26)
Hypertension	-1.18	0.14	-8.71	<.001	-1.18 (-1.44 ~ -0.91)
Disease duration, years	-0.01	0.00	-3.92	<.001	-0.01 (-0.09 ~ -0.01)
SBP, mmHg	-0.02	0.00	-5.18	<.001	-0.02 (-0.03 ~ -0.01)
WC, cm	-0.02	0.01	-3.79	<.001	-0.02 (-0.03 ~ -0.01)
Visceral-to-Subcutaneous fat ratio	-0.43	0.29	-1.49	0.137	-0.43 (-0.99 ~ 0.14)
PLT, *10^9/L	0.01	0.00	3.70	<.001	0.01 (0.01 ~ 0.01)
Hcy, µmol/L	-0.00	0.00	-0.49	0.625	-0.00 (-0.00 ~ 0.00)
eGFR, ml/min/1.73 m²	0.02	0.00	6.73	<.001	0.02 (0.01 ~ 0.02)
UACR, mg/g	-0.01	0.00	-2.05	0.040	-0.01 (-0.09 ~ -0.01)
BUN, mmol/L	-0.26	0.04	-6.86	<.001	-0.26 (-0.34 ~ -0.19)
Cr, µmol/L	-0.02	0.00	-5.69	<.001	-0.02 (-0.03 ~ -0.01)
TC, mmol/L	0.06	0.05	1.18	0.238	0.06 (-0.04 ~ 0.17)
LDL-c, mmol/L	0.09	0.08	1.15	0.252	0.09 (-0.06 ~ 0.24)
FFA, µmol/L	0.01	0.00	2.37	0.018	0.01 (0.01 ~ 0.01)
fT3, ng/L	0.03	0.03	0.96	0.338	0.03 (-0.03 ~ 0.10)
baPWV, cm/s	-0.01	0.00	-6.58	<.001	-0.01 (-0.09 ~ -0.01)
ABI	-1.54	0.55	-2.78	0.006	-1.54 (-2.63 ~ -0.45)
LVEF, %	0.04	0.01	3.16	0.002	0.04 (0.02 ~ 0.07)
DN	-0.52	0.14	-3.64	<.001	-0.52 (-0.80 ~ -0.24)
MAU	-0.41	0.16	-2.58	0.010	-0.41 (-0.73 ~ -0.10)
carotid plaque	-0.87	0.13	-6.46	<.001	-0.87 (-1.13 ~ -0.60)
Intermediate-to-high 10-year ASCVD risk	-1.92	0.20	-9.80	<.001	-1.92 (-2.31 ~ -1.54)

*p*-values < 0.05 were considered statistically significant.

In the multivariate stepwise linear regression model, older age, higher SBP, male sex, smoking, hypertension, and intermediate-to-high 10-year ASCVD risk were independently associated with impaired FMD (all *p* < 0.05) (**Table 5**).

**Table 5:** Multivariate stepwise linear regression analysis for FMD.

Variables	β	S.E	t	*p*	β (95%CI)
Age,years	-0.03	0.01	-3.83	<.001	-0.03 (-0.05 ~ -0.02)
Disease duration,years	0.00	0.00	0.16	0.870	0.00 (-0.00 ~ 0.00)
Male sex	-0.93	0.20	-4.63	<.001	-0.93 (-1.32 ~ -0.54)
Hypertension	-0.47	0.15	-3.03	0.002	-0.47 (-0.77 ~ -0.17)
Smoking	-0.51	0.17	-3.07	0.002	-0.51 (-0.84 ~ -0.18)
Drinking	-0.02	0.15	-0.14	0.888	-0.02 (-0.32 ~ 0.28)
SBP, mmHg	-0.01	0.00	-2.14	0.033	-0.01 (-0.02 ~ -0.01)
WC, cm	-0.01	0.01	-1.21	0.226	-0.01 (-0.02 ~ 0.00)
PLT, *10^9/L	-0.00	0.00	-0.16	0.872	-0.00 (-0.00 ~ 0.00)
FFA,µmol/L	0.00	0.00	0.80	0.424	0.00 (-0.00 ~ 0.00)
eGFR, ml/min/1.73 m²	0.00	0.00	0.95	0.340	0.00 (-0.00 ~ 0.01)
BUN, mmol/L	-0.08	0.05	-1.72	0.085	-0.08 (-0.17 ~ 0.01)
Cr, µmol/L	0.01	0.01	1.21	0.227	0.01 (-0.00 ~ 0.02)
UACR, mg/g	-0.00	0.00	-0.63	0.530	-0.00 (-0.00 ~ 0.00)
baPWV, cm/s	-0.00	0.00	-0.02	0.983	-0.00 (-0.00 ~ 0.00)
ABI	0.02	0.64	0.04	0.970	0.02 (-1.23 ~ 1.28)
DN	-0.20	0.14	-1.40	0.160	-0.20 (-0.48 ~ 0.08)
MAU	-0.16	0.18	-0.90	0.367	-0.16 (-0.50 ~ 0.19)
carotid plaque	-0.01	0.15	-0.06	0.954	-0.01 (-0.30 ~ 0.28)
Intermediate-to-high 10-year ASCVD risk	-0.83	0.24	-3.43	<.001	-0.83 (-1.31 ~ -0.36)

*p*-values < 0.05 were considered statistically significant.

### 3.5 Independent association between FMD and intermediate-to-high 10-year ASCVD risk

To further elucidate the relationship between FMD and cardiovascular risk, multivariate logistic regression was performed after adjusting for age, sex, SBP, hypertension, and smoking. The analysis demonstrated that for every 1% increase in FMD, the odds of having an intermediate-to-high 10-year ASCVD risk decreased by 10% (OR = 0.90, 95% CI: 0.82-0.99, *p* = 0.025) (**Table 6**).

**Table 6:** Multivariate logistic regression for intermediate-to-high 10-year ASCVD risk.

Variables	β	S.E	Z	*p*	OR (95%CI)
FMD	-0.10	0.05	-2.23	0.025	0.90 (0.82 ~ 0.99)
Male sex	0.27	0.28	0.95	0.341	1.31 (0.75 ~ 2.28)
Age	0.22	0.02	12.88	<.001	1.25 (1.20 ~ 1.29)
Hypertension	0.49	0.33	1.48	0.140	1.64 (0.85 ~ 3.14)
SBP	0.03	0.01	3.24	0.001	1.03 (1.01 ~ 1.04)
Smoking	0.75	0.27	2.77	0.006	2.11 (1.24 ~ 3.57)

*p*-values < 0.05 were considered statistically significant. OR, odds ratio; CI, confidence interval.

The receiver operating characteristic (ROC) curve analysis indicated that FMD exhibited moderate discriminative ability to identify participants classified as having intermediate-to-high 10-year ASCVD risk, with an area under the curve (AUC) of 0.700(95% CI:0.66-0.75) Using the maximum Youden index, the optimal cut-off value of FMD was 5.15, with a sensitivity of 0.67 (95%CI, 0.64-0.69) and specificity of 0.64 (95%CI, 0.57-0.72). A total of 1112 participants were classified as positive cases and 160 as negative cases in this analysis. (**Figure 2**).The restricted cubic spline (RCS) model showed a linear association between FMD and intermediate-to-high 10-year ASCVD risk (*p-*overall=0.004). No evidence of non-linearity was detected (*p-*non−linear=0.653), suggesting no statistically significant departure from linearity (**Figure 3**).

**Figure 2:**
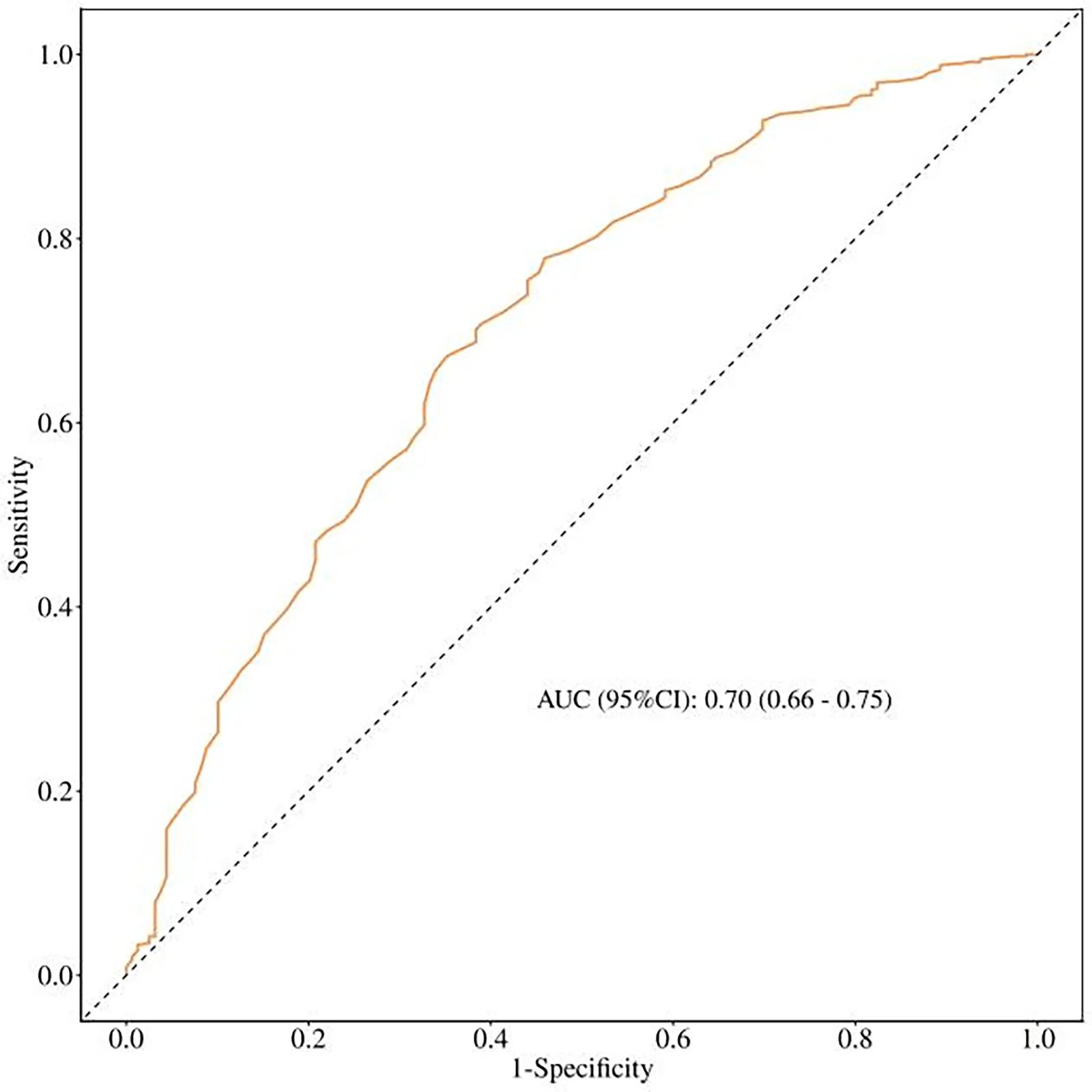
Receiver-operating characteristic (ROC) curve of FMD for identifying participants with intermediate-to-high 10-year ASCVD risk. The area under the curve (AUC = 0.700, 95% CI: 0.66-0.75). The optimal cut-off value determined by the maximum Youden index was 5.15, with a sensitivity of 0.67 (95%CI, 0.64-0.69) and specificity of 0.64 (95%CI, 0.57-0.72).

**Figure 3:**
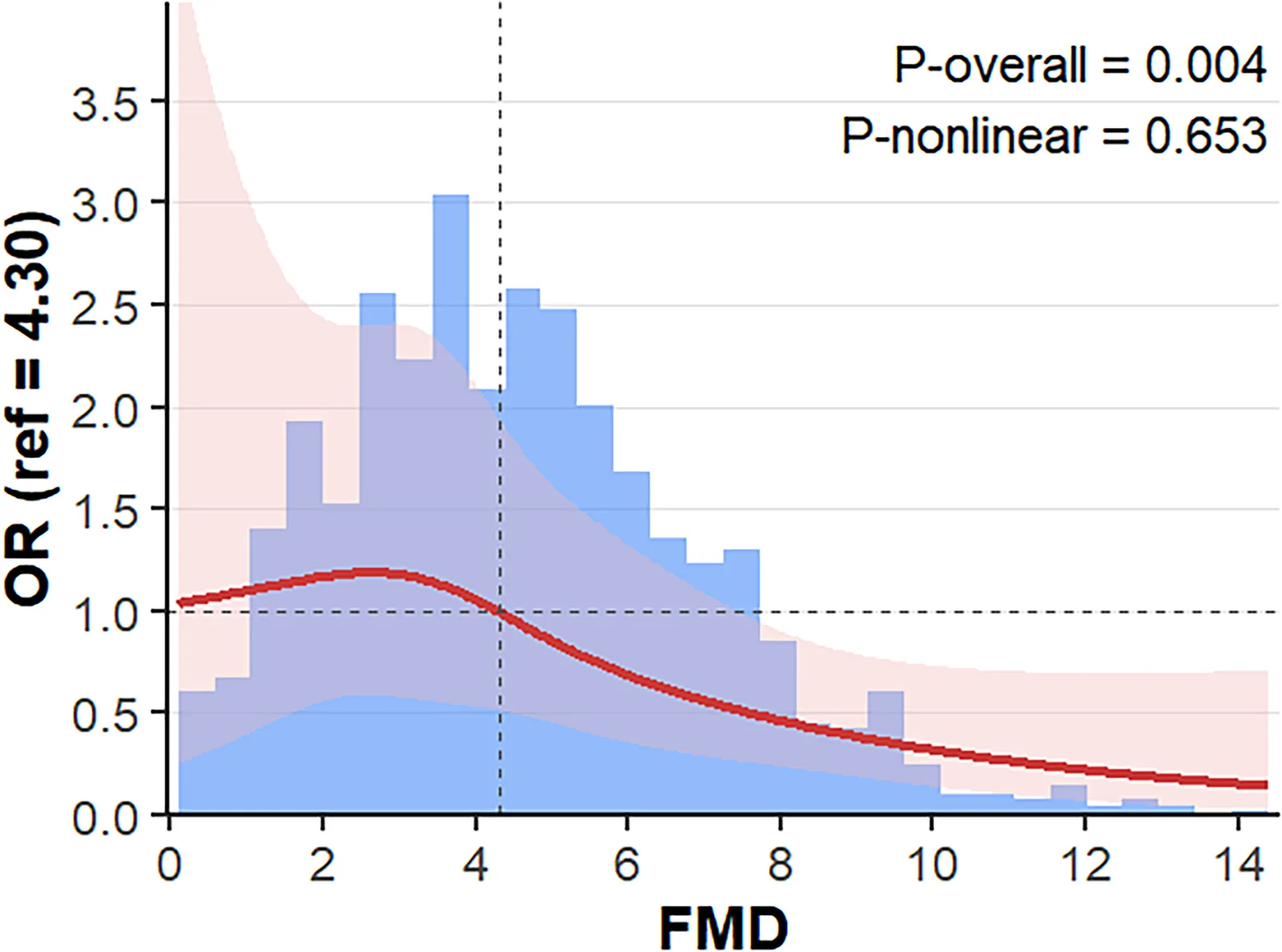
Restricted cubic spline (RCS) regression showed a linear association between FMD and the odds of intermediate-to-high estimated 10-year ASCVD risk across the observed FMD range. There was no statistically significant evidence for nonlinearity(p-overall = 0.004, p-non-linear = 0.653).Adjusted for age, sex, systolic blood pressure, hypertension and smoking.

## 4 Discussion

This large-scale retrospective study involving 1,272 patients with T2DM reveals a strikingly high prevalence of endothelial dysfunction (78.9%), defined as FMD < 6.5%. Our multivariate analyses identified older age, elevated systolic blood pressure, male sex, and smoking as independent determinants of impaired FMD. Crucially, we demonstrated for the first time in a large sample of Chinese patients with T2DM that FMD is independently and linearly associated with the intermediate-to-high 10-year risk of ASCVD. Furthermore, FMD exhibited moderate discriminative ability (AUC = 0.700, 95% CI: 0.66–0.75) for identifying individuals at intermediate-to-high 10-year ASCVD risk, suggesting its potential utility as a complementary tool to traditional risk scores.

Understanding the determinants of endothelial dysfunction in T2DM is of paramount clinical importance. Consistent with previous literature, our univariate analysis confirmed correlations between FMD and established cardiovascular risk factors, including age, smoking, alcohol consumption, blood pressure, sex, and waist circumference (15–17). Notably, FMD was also significantly associated with markers of diabetic complications, such as BUN, Cr, UACR, baPWV, ABI, eGFR, peripheral neuropathy, microalbuminuria, and carotid plaques (18–20). This aligns with the paradigm that endothelial dysfunction serves as a common pathophysiological substrate for both microvascular and macrovascular complications in diabetes (21). However, in our multivariate model adjusting for these confounders, only age, systolic blood pressure, male sex, and smoking remained independent predictors. This finding underscores the dominant role of hemodynamic stress and toxic exposures in driving endothelial injury in T2DM. Mechanistically, aging is associated with structural remodeling of endothelial cells and reduced nitric oxide (NO) bioavailability (22, 23). Tobacco smoke constituents (e.g., nicotine, aldehydes) directly induce endothelial apoptosis and vasospasm (24), while chronic hypertension increases wall shear stress, disrupting endothelial integrity (25). The higher susceptibility in males may be attributed to the loss of estrogen-mediated vascular protection, genetic predispositions, and distinct lifestyle patterns (26). These results reinforce the imperative for aggressive blood pressure control and smoking cessation as cornerstone strategies to preserve endothelial function and mitigate complication risks in T2DM.

A pivotal finding of this study is the linear negative dose-response relationship between FMD and intermediate-to-high 10-year ASCVD risk, as confirmed by restricted cubic spline (RCS) analysis (*p-*non-linear=0.653). This linearity association indicated that FMD was linearly related to the odds of intermediate-to-high 10-year ASCVD risk across the observed range, without a threshold effect. Pathophysiologically, endothelial dysfunction is the earliest detectable step in the atherosclerotic cascade (3). In T2DM, chronic hyperglycemia fuels oxidative stress and inflammation, impairing NO synthesis and promoting a pro-atherogenic state that progressively evolves into clinical ASCVD (21). Our observation shows that FMD was independently associated with the intermediate-to-high 10-year ASCVD risk (OR = 0.90 per 1% increase) even after adjusting for traditional risk factors. The moderate discriminative ability (AUC = 0.700, 95% CI: 0.66–0.75) reflects that FMD correlates with calculated ASCVD risk burden in T2DM patients. We hypothesize that FMD may offer supplementary clinical information for T2DM patients with discordant risk profiles (e.g., low traditional risk but significantly impaired FMD). Further prospective outcome-driven investigations are needed to test whether FMD-guided risk evaluation can support earlier intensive lifestyle and pharmacologic primary-preventive strategies.

Several limitations of this study should be acknowledged. First, the retrospective design precludes causal inference; we cannot definitively conclude that endothelial dysfunction causes the elevated risk, nor can we validate FMD’s ability to predict hard cardiovascular events (e.g., MI, stroke) over time. Second, as a single-center study in Zhejiang, China, the generalizability to other ethnic populations requires validation. Third, we did not assess the impact of interventions targeting endothelial function on subsequent risk reduction. Fourth, our binary outcome was a China-PAR-computed risk category rather than observed clinical events. Derived from covariates used in our regressions, this score only allows us to assess associations rather than FMD’s incremental value for real ASCVD events. Future multicenter, prospective cohort studies with long-term follow-up are needed to elucidate the molecular mechanisms linking FMD to ASCVD events and to evaluate whether FMD-guided therapy improves cardiovascular outcomes in T2DM. Finally, Intermediate-to-high 10-year ASCVD risk was defined as the binary outcome for primary regression, ROC, and restricted cubic spline analyses in the full cohort; no separate analyses were performed for low-risk participants. Despite these limitations, our study benefits from a large sample size, standardized data collection via the National MMC protocol, and the robust application of RCS modeling to characterize the FMD-ASCVD relationship.

## 5 Conclusions

Endothelial dysfunction is highly prevalent in patients with T2DM, driven primarily by age, hypertension, male sex, and smoking. Importantly, FMD exhibits an independent, linear inverse association with intermediate-to-high 10-year ASCVD risk and shows moderate discriminative ability for this computed risk grouping. Our findings encourage further exploration of FMD assessment in T2DM patients. Whether combining FMD with traditional risk scores could enable more refined risk stratification, early identification of high-risk individuals and timely targeted interventions to curb the progression of atherosclerotic cardiovascular disease remains to be confirmed in prospective studies with hard ASCVD endpoints.

## Data Availability

The raw data supporting the conclusions of this article will be made available by the authors, without undue reservation.

## Glossary

ABI: Ankle-brachial index
ALT: alanine aminotransferase
APOA-1: apolipoprotein A-1
APOB: apolipoprotein B
AST: aspartate aminotransferase
BMI: body mass index
BUN: blood urea nitrogen
CAD: coronary artery disease
Cr: creatinine
DBP: diastolic blood pressure
DN: diabetic nephropathy
DR: diabetic retinopathy
eGFR: estimated glomerular filtration rate
FBG: fasting blood-glucose
F-C-P: fasting C-peptide
FFA: free fatty acids
FINS: fasting insulin
FMD: flow-mediated dilation
fT3: free triiodothyronine
fT4: free thyroxine
HbA1c: glycated hemoglobin
Hb: hemoglobin
Hcy: homocysteine
HDL-c: High-density lipoprotein-cholesterol
HLP: hyperlipidemia
HOMA-IR: homeostasis model assessment of insulin resistance
HOMA-β: homeostasis model assessment of β-cell function
HTN: hypertension
HUA: hyperuricemia
LVEF: left ventricular ejection fraction
LDL-c: Low-density lipoprotein-cholesterol
MAU: microalbuminuria
NAFLD: non-alcoholic fatty liver disease
PAD: peripheral artery disease
PLT: platelet count
RBC: red blood cell count
SBP: systolic blood pressure
SFA: subcutaneous fat area
TC: total cholesterol
TG: triglycerides
TSH: Thyroid-stimulating hormone
T2DM: type 2 diabetes mellitus
UA: uric acid
UACR: urinary albumin-to-creatinine ratio
VFA: visceral fat area
WBC: white blood cell count
WC: waist circumference
2h-C-P: 2-hour postprandial C-peptide
2h-INS: 2-hour postprandial insulin
2h-PG: 2-hour postprandial plasma glucose
γ-Gt: Gamma-glutamyl transferase
10-year ASCVD risk: 10-year risk of atherosclerotic cardiovascular disease.

## References

[1] Zheng Y Ley SH Hu FB . Global aetiology and epidemiology of type 2 diabetes mellitus and its complications. Nat Rev Endocrinol. (2018) 14:88–98. doi: 10.1038/nrendo.2017.151 29219149

[2] Xu S Ilyas I Little PJ Li H Kamato D Zheng X . Endothelial dysfunction in atherosclerotic cardiovascular diseases and beyond: From mechanism to pharmacotherapies. Pharmacol Rev. (2021) 73:924–67. doi: 10.1124/pharmrev.120.000096 34088867

[3] Gimbrone MA García-Cardeña G . Endothelial cell dysfunction and the pathobiology of atherosclerosis. Circ Res. (2016) 118:620–36. doi: 10.1161/CIRCRESAHA.115.306301 26892962 PMC4762052

[4] Heiss C Rodriguez-Mateos A Bapir M Skene SS Sies H Kelm M . Flow-mediated dilation reference values for evaluation of endothelial function and cardiovascular health. Cardiovasc Res. (2023) 119:283–93. doi: 10.1093/cvr/cvac095 35709326

[5] van Sloten TT Henry RMA Dekker JM Nijpels G Unger T Schram MT . Endothelial dysfunction plays a key role in increasing cardiovascular risk in type 2 diabetes: the Hoorn study. Hypertension. (2014) 64:1299–305. doi: 10.1161/hypertensionaha.114.04221 25225211

[6] Matsuzawa Y Kwon T Lennon RJ Lerman LO Lerman A . Prognostic value of flow‐mediated vasodilation in brachial artery and fingertip artery for cardiovascular events: A systematic review and meta‐analysis. JAHA. (2015) 4:e002270. doi: 10.1161/JAHA.115.002270 26567372 PMC4845238

[7] Yeboah J Crouse JR Hsu F-C Burke GL Herrington DM . Brachial flow-mediated dilation predicts incident cardiovascular events in older adults: The Cardiovascular Health Study. Circulation. (2007) 115:2390–7. doi: 10.1161/CIRCULATIONAHA.106.678276 17452608

[8] Lloyd-Jones DM Braun LT Ndumele CE Smith SC Sperling LS Virani SS . Use of risk assessment tools to guide decision-making in the primary prevention of atherosclerotic cardiovascular disease: A special report from the American Heart Association and American College of Cardiology. Circulation. (2019) 139:e1162–77. doi: 10.1161/CIR.0000000000000638 30586766

[9] ElSayed NA McCoy RG Aleppo G Balapattabi K Beverly EA Briggs Early K . 2. Diagnosis and classification of diabetes: Standards of care in diabetes—2025. Diabetes Care. (2025) 48:S27–49. doi: 10.2337/dc25-S002 39651986 PMC11635041

[10] Matthews DR Hosker JP Rudenski AS Naylor BA Treacher DF Turner RC . Homeostasis model assessment: insulin resistance and ?-cell function from fasting plasma glucose and insulin concentrations in man. Diabetologia. (1985) 28:412–9. doi: 10.1007/BF00280883 3899825

[11] Levey AS Stevens LA Schmid CH Zhang Y Castro AF Feldman HI . A new equation to estimate glomerular filtration rate. Ann Intern Med. (2009) 150:604–12. doi: 10.7326/0003-4819-150-9-200905050-00006 19414839 PMC2763564

[12] Corretti MC Anderson TJ Benjamin EJ Celermajer D Charbonneau F Creager MA . Guidelines for the ultrasound assessment of endothelial-dependent flow-mediated vasodilation of the brachial artery. J Am Coll Cardiol. (2002) 39:257–65. doi: 10.1016/S0735-1097(01)01746-6 11788217

[13] Yang X Li J Hu D Chen J Li Y Huang J . Predicting the 10-year risks of atherosclerotic cardiovascular disease in Chinese population: The China-PAR Project (Prediction for ASCVD Risk in China). Circulation. (2016) 134:1430–40. doi: 10.1161/CIRCULATIONAHA.116.022367 27682885

[14] Yang X Chen J Li J Cao J Lu X Liu F . Risk stratification of atherosclerotic cardiovascular disease in Chinese adults. Chronic Dis Trans Med. (2016) 2:102–9. doi: 10.1016/j.cdtm.2016.10.001 29063030 PMC5643596

[15] Skaug E-A Madssen E Aspenes ST Wisløff U Ellingsen Ø . Cardiovascular risk factors have larger impact on endothelial function in self-reported healthy women than men in the HUNT3 Fitness Study. PLoS One. (2014) 9:e101371. doi: 10.1371/journal.pone.0101371 24991924 PMC4081583

[16] Holder SM Bruno RM Shkredova DA Dawson EA Jones H Hopkins ND . Reference intervals for brachial artery flow-mediated dilation and the relation with cardiovascular risk factors. Hypertension. (2021) 77:1469–80. doi: 10.1161/HYPERTENSIONAHA.120.15754 33745297

[17] Montazeri Z Hashemi-Madani N Iraji H Sohrabi M Alaei-Shahmiri F Emami Z . Non-alcoholic fatty liver disease and compromised endothelial function in people with type 2 diabetes. BMC Endocr Disord. (2023) 23:202. doi: 10.1186/s12902-023-01460-w 37749528 PMC10518908

[18] Koyoshi R Hitaka-Yoshimine Y Shiga Y Kuwano T Sugihara M Ike A . Associations between microalbuminuria and parameters of flow-mediated vasodilatation obtained by continuous measurement approaches. Clin Exp Hypertension. (2018) 40:715–20. doi: 10.1080/10641963.2018.1425422 29351006

[19] Roustit M Loader J Deusenbery C Baltzis D Veves A . Endothelial dysfunction as a link between cardiovascular risk factors and peripheral neuropathy in diabetes. J Clin Endocrinol Metab. (2016) 101:3401–8. doi: 10.1210/jc.2016-2030 27399351 PMC5010566

[20] Tomiyama H Ishizu T Kohro T Matsumoto C Higashi Y Takase B . Longitudinal association among endothelial function, arterial stiffness and subclinical organ damage in hypertension. Int J Cardiol. (2018) 253:161–6. doi: 10.1016/j.ijcard.2017.11.022 29174285

[21] Yang D-R Wang M-Y Zhang C-L Wang Y . Endothelial dysfunction in vascular complications of diabetes: a comprehensive review of mechanisms and implications. Front Endocrinol. (2024) 15:1359255. doi: 10.3389/fendo.2024.1359255 38645427 PMC11026568

[22] Tracy EP Hughes W Beare JE Rowe G Beyer A LeBlanc AJ . Aging-induced impairment of vascular function: mitochondrial redox contributions and physiological/clinical implications. Antioxid Redox Signal. (2021) 35:974–1015. doi: 10.1089/ars.2021.0031 34314229 PMC8905248

[23] Gogulamudi VR Cai J Lesniewski LA . Reversing age-associated arterial dysfunction: insight from preclinical models. J Appl Physiol (1985). (2018) 125:1860–70. doi: 10.1152/japplphysiol.00086.2018 29745797 PMC6442667

[24] Münzel T Hahad O Kuntic M Keaney JF Deanfield JE Daiber A . Effects of tobacco cigarettes, e-cigarettes, and waterpipe smoking on endothelial function and clinical outcomes. Eur Heart J. (2020) 41:4057–70. doi: 10.1093/eurheartj/ehaa460 32585699 PMC7454514

[25] Cheng CK Wang N Wang L Huang Y . Biophysical and biochemical roles of shear stress on endothelium: a revisit and new insights. Circ Res. (2025) 136:752–72. doi: 10.1161/CIRCRESAHA.124.325685 40146803 PMC11949231

[26] Arnold AP Cassis LA Eghbali M Reue K Sandberg K . Sex hormones and sex chromosomes cause sex differences in the development of cardiovascular diseases. ATVB. (2017) 37:746–56. doi: 10.1161/ATVBAHA.116.307301 28279969 PMC5437981

